# A Quantitative Assessment of the Rush Hour of Life in Austria, Italy and Slovenia

**DOI:** 10.1007/s10680-018-9502-4

**Published:** 2018-11-21

**Authors:** Marina Zannella, Bernhard Hammer, Alexia Prskawetz, Jože Sambt

**Affiliations:** 1 Department of Methods and Models for Economics, Territory and Finance, “Sapienza” University of Rome, Via del Castro Laurenziano n.9, 00161 Rome, Italy; 2 Wittgenstein Centre for Demography and Global Human Capital (IIASA, VID/ÖAW, WU), Welthandelsplatz 2/Level 2, 1020 Vienna, Austria; 3 Faculty of Economics, University of Ljubljana, Kardeljeva Ploscad 17, 1000 Ljubljana, Slovenia; 4 Institute of Statistics and Mathematical Methods in Economics, TU Wien, Vienna, Austria

**Keywords:** Gender, Life course, Time use, Total work, Leisure inequality, Breadwinner models

## Abstract

This article builds on time use data to explore cross-country differences between Austria, Italy and Slovenia in unpaid labour and its implications in terms of gender distribution of total work. A contribution of this paper is to measure the ‘rush hour of life’ (RHOL) based on age spans in which individuals’ working time (including paid and unpaid work) exceeds their free time. In total, men and women work similar hours in Austria, whereas Italy and Slovenia show a gender gap with women working an average of approximately 50 min more per day during prime working ages. The different compositions and loads of total work are reflected in cross-country variations of the length and intensity of the RHOL, with Slovenian women reporting, on average, the larger squeeze of time. However, breadwinner arrangements differ considerably among the three countries, which can affect the amounts of work and free time available for men and even more so for women. Therefore, we further extend our analysis by developing a regression model to quantitatively assess the association between couples’ working arrangements and levels of the RHOL indicator for men and women. Results indicate a dual burden for women in dual-earner couples, squeezing out their free time. By contrast, women in male-breadwinner arrangements report the lowest amounts of total work. Breadwinner models show no significant relation to male levels of work and free time, with the main exception of Italy where men face higher RHOL in full-time employed couples.

**Electronic supplementary material:**

The online version of this article (10.1007/s10680-018-9502-4) contains supplementary material, which is available to authorized users.

## Introduction

The last 50 years have brought far-reaching changes to societies, individuals and family lives. Women’s epochal entry into the labour market, the emergence of atypical employment, the increasing complexity of individual life paths and the rise of diverse family types contributed to change the rhythm of our lives. Work–family reconciliation has become a core issue of contemporary welfare state policies since women, or ‘the nation’s unpaid caregivers’ (Bianchi 2011), have been incorporated in the labour market entailing a reorganisation of family times. What happened after this revolution? This is the question that scholars in social sciences have been asking themselves over the last decades. Are families ‘overworked’ (Jacobs and Gerson 2001)? Are family and paid-work obligations equally distributed among the genders or have social changes brought a new form of inequality, i.e. leisure inequality?

Answering these questions is of fundamental relevance for today’s societies. Ineffective institutional response to competing work and family time demands can result in high costs for individuals and societies at large, such as loss of female market work and their lower investments in education and career, increasing risk of poverty for households with children, and low fertility (Torres et al. 2007).

This article contributes to the analysis of work–life imbalances by exploring gender differences in the composition and distribution of total work (i.e. the combination of paid and unpaid work) and free time available from a life course and comparative perspective. Our main objective is to quantitatively assess the rush hour of life (RHOL) and its cross-country variations for men and women in different breadwinner arrangements (i.e. couples’ internal organisation of paid and unpaid work). We conduct our analysis for three different countries: Austria, Italy and Slovenia, using original micro-data from national time use surveys (TUS). The choice of the countries is mainly motivated by the existence of substantial differences in their welfare arrangements. Austria and Italy are two examples of conservative welfare regimes with the family considered to be responsible for intergenerational obligations (Esping-Andersen 1990). Nevertheless, the two countries show interesting dissimilarities: in Italy male-breadwinner arrangements are quite frequent among couples with children, whereas the *modified breadwinner model* (Haas 2005), with one person working full time and the other part time, prevails among Austrian parents (see Zagheni et al. 2015). Saraceno and Keck (2011) identified Austria as a form of *supported familialism* where policies (mostly through financial transfers) actively support women in assuming the main responsibilities for caring needs of the family and encourage mothers to remain in the labour market by ensuring extended job protection over long parental-leave periods, whereas, according to the authors, the Italian welfare system can be seen as a *familialism by default,* i.e. the shift of intergenerational responsibilities to families goes along with minimal or absent policy and financial support from the state. The situation is very different for Slovenia where, as a historical legacy, the dual-earner couple is the norm. Slovenia clearly supports female participation in the formal economy, although it is less clear whether the gender egalitarian ideology is extended to the household sphere and to kin obligations or whether it is limited to the market boundaries. For instance, paternal leave is not common among Slovenian fathers, labour market flexibilities such as part-time work are rare—all things which may suggest the existence of a dual burden for women.

This paper is structured as follows. After giving an overview of the relevant literature, we describe the data sources and provide descriptive statistics of the age profiles of the use of time for different activities. By doing that, we obtain a first broad picture of gender differences in time use over the life course across the three countries. In the following section, we measure and analyse total labour, consisting of paid and unpaid work, by age and gender with a focus on non-market work. Having the detailed TUS micro-data available, we can analyse the different components of unpaid work as well as differences across the three countries. We thereby gain insight into the division of market and non-market work across age groups related to different life course stages and genders. Then, we analyse the allocation of people’s time between labour and free time over the life cycle. We present our measures of the RHOL, defined as the age span during which average working time exceeds free time (leisure and personal care). The RHOL is especially intense at ages during which individuals usually combine work and family responsibilities. There are considerable differences among countries: men and women work similar hours in Austria, whereas Italian and Slovenian women face a more pronounced RHOL compared to men. However, our indicator of the RHOL is expressed as age- and sex-specific averages and thus gives only a rough measure of the time squeeze over the life course and of the related gender differences among the three countries. In particular, compositional effects due to different breadwinner arrangements may significantly affect the amounts of work and free time available for men and women in the three countries. Therefore, we further extend our analysis by focusing on couples and using multivariate statistical analysis to identify the relation between different breadwinner arrangements and the RHOL indicator for men and women when all other relevant characteristics of the individuals are controlled for. The final section concludes.

## Background Literature

About one decade after women’s massive entry into the labour market, Smith (1979) called for a ‘subtle revolution’ of gender roles and society at large. Smith looked with a fair optimism to the ability of both families and society to gradually adapt to the new role of women and relax traditional gender roles and stereotypes. Exactly 10 years later, Hochschild (1989) coined the term *stalled revolution* to indicate that the women’s revolutionary entry into the market had not been accompanied by a similar revolutionary entry of men into the household: despite men’s increasing participation in domestic work, women still bear the main responsibility for caregiving in the eyes of society and of families.

Among other main societal changes that have characterised the last decades affecting individual and family’s time allocation, it is worthy to recall the rise of atypical work together with non-standard working hours and a cultural shift towards intensive parenting (e.g. Bianchi and Milkie 2010; Gauthier et al. 2004). A body of research documented an increase in average working time as well as of non-standard working hours (e.g. evening or weekend work hours) occurring in parallel with the emergence of the dual-earner middle-class norm (Schor 1991). Gershuny (2000) suggested a historical reversal in the relation between leisure and social status: from the ‘leisure class’ era when the consumption of leisure time was associated with social prestige to today’s societies where being busy is associated with high social status. Beside the growth in paid work for both genders, time investment in childrearing of mothers and fathers has increased as compared to the 1960s when mothers still had the sole role of providing care (Sayer et al. 2004). Sociological and ethnographic studies have shown that intensive childrearing (Craig et al. 2014) or ‘concerted cultivation’ (Lareau 2002), i.e. conspicuous and diversified parental time investment, help children to develop important life skills and thus to enhance their future possibilities. Contemporary employed mothers devote similar hours to childcare as the ‘golden era housewives’. Time use studies show evidence for employed mothers subtracting time from leisure and sleep to meet their job and maternal responsibilities (Bianchi 2011). Fathers in dual-earner couples are more likely to participate in childcare, especially when women work non-standard hours (Presser 1989). Delayed marriage and childbearing are likely to create a situation where individuals have to face multiple responsibilities and time-consuming tasks, such as caring for young children, building their careers and setting up their home.

All these changes have contributed to the emergence of objective time scarcity for families and individuals—and the subjective feeling of being rushed. Research has documented that working couples with young children are more likely to feel time-squeezed (e.g. Craig and Brown 2017). Cross-national time use studies have shown a universal negative relation between having preschool children and parental leisure time (e.g. Anxo et al. 2011). The emergence of intensive parenting norms has contributed to increase the magnitude of the leisure squeeze for parents over the last decades; however, the decline in free time has been sharper for mothers than for fathers (Sayer 2005). The permanence of women as main caregivers together with their shift into the market has raised concern for the emergence of leisure inequality between genders (e.g. Fox and Nickols 1983). Nonetheless, a number of studies reported that, considering the combination of market and family work, men and women are working similar hours in total (e.g. Bianchi 2011). Burda et al. (2013) suggested that ‘iso-work’, i.e. a similarity in total hours worked by men and women, exists in rich non-Catholic countries, whereas women have a higher workload in Catholic countries. A recent article confirmed that iso-work does not hold in predominantly Catholic countries and suggested the existence of specific time use patterns for Mediterranean countries, where stringent gender roles persist mostly due to social norms (Gimenez-Nadal and Sevilla 2014). Despite reporting an overall trend towards narrowing gender differences in time allocation to paid and unpaid work, Sayer (2005) provided evidence for women continuing to work more in the household and having about 30 min less leisure time per day than men.

This article builds on time use data to explore gender differences among Austria, Italy and Slovenia in unpaid labour and their implication on the distribution of total workload between genders over the life course. We develop a measure of the rush hour of life, defined as the age span during which the combination of familiar and professional responsibilities results in large amounts of total work and, hence, little disposable time for leisure and personal care. Existing literature has identified *working*-*time regimes* that shape the work–life balance of men and women over the life course (Burgoon and Baxandall 2004; Torres et al. 2007). Working-time regimes emerge through a combination of levels of employment and unemployment, regulation of working hours, public-care availability and policies supporting female labour force participation. It is a widely shared opinion that the family–work conflict has become increasingly problematic with the emergence of dual-earner families. Therefore, we further develop our analysis to empirically assess the relation between different couples’ breadwinner arrangements and the RHOL of men and women. Among the other characteristics related to the RHOL, the presence of preschool children is of particular interest.

To our knowledge, this is the first attempt to quantitatively assess the rush hour of life for men and women across the life course. The work–family conflict often experienced during the RHOL may affect several aspects of individuals’ and households’ lives including investments in education, career perspectives, earnings and fertility decisions. All these factors, in turn, can expose individuals to increasing risk of poverty and consequences entailed by low investment in human capital and by low fertility levels. Measuring the RHOL and analysing its association with different work–family arrangements and other relevant household and individual characteristics therefore is inherently relevant for today’s societies.

## Data

Our analysis builds upon the time use survey data conducted by national statistical offices in 2008 (Austria and Italy) and 2000 (Slovenia). Unfortunately, there is no more recent time use survey available for Slovenia, so we have to keep this time difference of 8 years between the Slovenian and Austrian/Italian surveys in mind when interpreting the results. The sample size was 8232/44,606/6190 individuals and 4757/18,250/2364 households for Austria/Italy/Slovenia, respectively. In Slovenia, each respondent recorded his/her activities on two randomly selected days, of which one was a week day (from Monday to Friday) and the other a weekend day (Saturday or Sunday). In Italy, diaries were filled in by all members of the household aged 3 years and over,^1^ while in Slovenia and Austria only persons aged 10 years and over were interviewed.^2^ To be comparable across countries, we restrict our analysis for Italy to persons aged 10+.^3^

A first step in our analysis consists in distinguishing the activities which classify as unpaid work from other activities on which people spend time. Our guideline is the third-party criterion (Reid 1934), according to which an activity counts as work if you could pay someone else to do it for you. This criterion excludes work from non-work activities like sleeping, socialising with friends, etc.

We group activities from the surveys to meaningful larger groups that are in line with the Harmonised European Time Use Surveys (HETUS, Eurostat 2009) classification and the purpose of our analysis. A large majority of unpaid work is taking place within households. These activities are presented as ‘housework’ including activities related to cooking, cleaning, doing laundry, shopping, gardening and pet care, construction and repair and a few more remaining activities. We will present a detailed decomposition of housework by those activities later, and here we separately present ‘childcare, adult care’ and ‘voluntary’^4^ work. The category ‘paid work’ covers all activities related to employment for remuneration and thus includes working time at the main job and a possible second job (at home) but also travel to/from work, job search and breaks during the working time.^5^ ‘Education’ refers to time devoted to both formal and informal studies. The main component of ‘personal care’ is sleeping, but it includes also lying sick in bed, washing and dressing. Finally, ‘leisure’ comprises activities related to sports, hobbies, games, mass media, social life activities, cultural events, relaxing, etc.

Table 1 gives a general overview on average time use across the various activities by age and gender in the three countries (the related estimates of the standard errors are reported in Table A2 in Online Appendix). On average, people spend most of their time (about 11 h per day) in personal care activities such as sleeping and eating. Other important activities are leisure, paid work and housework. The amount of time that is on average devoted to these activities strongly depends on the age and sex of individuals. Rather little time is devoted to adult care and voluntary work: on average between 3 and 5 min per day to adult care and between 5 and 11 min to voluntary work.

**Table 1:** Average time use by age, gender and activity in minutes per day

	Austria—men					Austria—women				
	10–24	25–39	40–59	60+	Total	10–24	25–39	40–59	60+	Total
Unpaid work	*60*	*134*	*150*	*224*	*143*	*108*	*296*	*285*	*318*	*263*
*Housework*	*52*	*94*	*127*	*206*	*120*	*88*	*198*	*253*	*299*	*222*
*Childcare*	*3*	*33*	*13*	*5*	*14*	*14*	*91*	*22*	*9*	*33*
*Adult care*	*1*	*1*	*2*	*2*	*2*	*1*	*2*	*5*	*4*	*3*
*Voluntary*	4	6	8	11	7	5	5	5	6	5
Paid work	162	411	360	38	260	128	248	220	13	154
Education	203	10	4	2	47	193	16	5	2	41
Personal care	658	593	622	732	647	676	625	640	725	667
Leisure	354	288	299	439	339	332	252	285	379	311
Other/unknown	3	4	5	5	4	3	3	5	3	4
Total	1440	1440	1440	1440	1440	1440	1440	1440	1440	1440

	Italy—men					Italy—women				
	10–24	25–39	40–59	60+	Total	10–24	25–39	40–59	60+	Total
Unpaid work	31	84	120	190	115	90	312	359	348	303
*Housework*	*26*	*55*	*94*	*166*	*92*	*75*	*225*	*316*	*324*	*260*
*Childcare*	*1*	*23*	*17*	*6*	*13*	*9*	*80*	*27*	*8*	*30*
*Adult care*	*2*	*2*	*4*	*7*	*4*	*1*	*2*	*8*	*6*	*5*
*Voluntary*	*2*	*4*	*5*	*11*	*6*	*5*	*5*	*8*	*10*	*8*
Paid work	90	392	371	55	246	57	212	183	14	118
Education	232	14	1	1	44	240	19	2	1	43
Personal care	700	669	659	742	689	709	672	659	730	691
Leisure	386	280	287	451	345	342	223	236	346	283
Other/unknown	1	1	2	1	1	2	2	1	1	2
Total	1440	1440	1440	1440	1440	1440	1440	1440	1440	1440

	Slovenia—men					Slovenia—women				
	10–24	25–39	40–59	60+	Total	10–24	25–39	40–59	60+	Total
Unpaid work	82	163	193	242	169	119	307	330	358	286
*Housework*	*69*	*121*	*169*	*216*	*143*	*100*	*225*	*307*	*336*	*249*
*Childcare*	*2*	*29*	*7*	*10*	*12*	*13*	*73*	*13*	*12*	*28*
*Adult care*	*2*	*3*	*4*	*3*	*3*	*2*	*3*	*4*	*5*	*4*
*Voluntary*	*9*	*10*	*13*	*13*	*11*	*4*	*6*	*6*	*5*	*5*
Paid work	89	352	288	53	209	67	249	212	22	145
Education	193	15	2	1	50	210	17	4	1	50
Personal care	669	611	625	695	647	677	619	624	698	652
Leisure	400	293	326	444	359	362	242	265	357	302
Other/unknown	7	6	6	5	6	5	6	5	4	5
Total	1440	1440	1440	1440	1440	1440	1440	1440	1440	1440

Source: Authors’ calculations on time use surveys in Austria (2008), Italy (2008) and Slovenia (2000)

The values presented in Table 1 are averages for the population. However, not all individuals devoted time to all groups of activities on the day they recorded their time use and filled out the time diary. A decomposition of the average number of minutes spent on different activities into (a) the share of individuals involved in a certain activity and (b) the average time spent on the activity out of all those who were involved in that activity is found in Table A3 in Online Appendix.

## Total Work

**Fig. 1:**
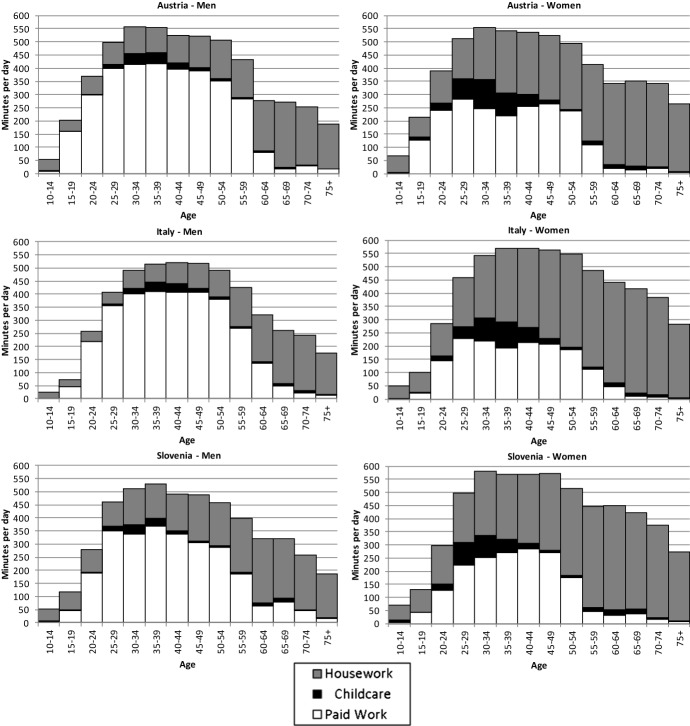
Work activities by age and gender in minutes per day. *Source*: Authors’ calculations on time use surveys in Austria (2008), Italy (2008) and Slovenia (2000). *Note* We present childcare separately from other unpaid work

Figure 1 plots the average time used for work activities (paid work, childcare and other unpaid work) by age and gender for the three countries. There are remarkable cross-country differences in the total amount of time devoted to work, as well as in its distribution between men and women.

Slovenian men devote a lower amount of time to paid work compared to men in Austria and Italy. While Austrian and Italian men at age 30–49 spend on average about 400 min per day on paid work, the corresponding value for Slovenian men is only about 350 min. However, Slovenian men compensate less paid work by providing more unpaid work compared to Austrian and Italian men. Consequently, they work in total similar hours as Italian men, but still lagging slightly behind the Austrian men.

Italian women devote on average little time to paid work: slightly above 200 min per day in the age group from 30 to 49, compared to about 250 min in Austria and 270 min in Slovenia. However, Italian women at these ages devote much more time to housework as compared to Austrian women and slightly more than Slovenian women. The average time that women aged 30–49 devote to total work is therefore similar in Italy and Austria, about 550 min per day, while in Slovenia it is slightly higher. The qualitative pattern of paid and unpaid work during the working age differs across countries. In Austria and Italy, women often reduce the amount of paid work when having young children—either in the form of part-time work or withdrawing from the labour market for several years. In Slovenia, on the other hand, women usually return to full-time employment after 1 year of parental leave. However, Slovenian women retire distinctively earlier than Austrian and Italian women. The average total amount of work is very stable between age 30 and 49 in all countries, despite different levels of paid work and changing shares of paid versus unpaid work.

During the working age, in Austria the total amount of work is about the same for both genders, whereas Italy and Slovenia show a gender gap in total work of about 50 min per day. Our results are in line with the previous literature. Burda et al. (2013) document that Italian women work on average 40 min longer per day than Italian men. The authors claim that the iso-work phenomenon does not hold in predominantly Catholic countries, proposing social norms as an explanation. Our results suggest that this explanation does not fit in the cases of Austria and Slovenia. Austria, though a Catholic country, reported similar average amounts of total labour for men and women due to the existence of gender specialisation. In Slovenia, the amount of women’s paid work is closer to that of men than in the other two countries, which reflects the historical legacy of a socialist system striving for equality—including gender equality. Nevertheless, our results indicate that gender equality does not hold for unpaid work. After retirement, men start to devote more time to household and family care. However, in all the three countries women continue, on average, to provide much more unpaid work than men. Therefore, the latter are left with more time for leisure and personal care.

Because there are large differences in the three countries with regard to the average amount of time devoted to housework, it is interesting to have a closer look at the activities in the category ‘housework’ in Table 2. In all three countries, the average amount and type of housework carried out by men is rather similar, except for gardening: Slovenian men devote considerably more time to gardening and pet care than Austrian and Italian men. In all three countries, women allocate much more time to housework activities than men; the total amount is rather similar in Slovenia and Italy, with an average of 249 and 260 min, respectively. Slovenian women devote more time to cooking and gardening, whereas—in line with previous studies (Burda et al. 2008)—Italian women spend more time on cleaning than Slovenian and Austrian women. With an average of around 222 min, the latter devote less time to housework than women in the other two countries, which is explained by the much lower amount of time Austrian women spend on cooking and a somewhat lower amount of time spent on cleaning activities.

**Table 2:** Further decomposition of the ‘housework’ category from Table 1: average time use by age, gender and activity in minutes per day

	Austria—men					Austria—women				
	10–24	25–39	40–59	60+	Total	10–24	25–39	40–59	60+	Total
Cooking	10	18	23	30	20	21	62	78	99	69
Cleaning	13	20	28	45	27	20	48	59	63	50
Laundry	0	3	4	5	3	6	26	35	40	29
Shopping	17	22	27	44	27	25	37	39	44	37
Gardening/pet care	5	11	23	51	23	9	19	33	44	28
Construction/repair	6	17	18	22	16	3	2	3	2	3
Other	1	3	4	9	4	4	4	6	7	6
Total	52	94	127	206	120	88	198	253	299	222

	Italy—men					Italy—women				
	10–24	25–39	40–59	60+	Total	10–24	25–39	40–59	60+	Total
Cooking	6	14	20	32	19	25	84	118	127	99
Cleaning	6	11	16	25	15	24	75	101	98	82
Laundry	0	0	1	1	1	3	20	34	40	28
Shopping	10	21	32	48	30	21	41	53	46	43
Gardening/pet care	3	5	18	48	20	2	4	9	12	7
Construction/repair	1	3	5	6	4	0	0	0	0	0
Other	0	1	2	6	3	0	1	1	1	1
Total	26	55	94	166	92	75	225	316	324	260

	Slovenia—men					Slovenia—women				
	10–24	25–39	40–59	60+	Total	10–24	25–39	40–59	60+	Total
Cooking	9	15	24	27	19	32	90	124	146	101
Cleaning	19	23	33	45	30	29	49	61	61	52
Laundry	0	1	1	3	1	6	27	37	38	28
Shopping	11	20	28	29	22	18	31	34	27	28
Gardening/pet care	20	34	57	79	47	13	24	45	60	36
Construction/repair	8	25	22	28	21	1	2	3	1	2
Other	2	3	4	5	3	1	2	3	3	2
Total	69	121	169	216	143	100	225	307	336	249

Source: Authors’ calculations on time use surveys in Austria (2008), Italy (2008) and Slovenia (2000)

## The ‘Rush Hour of Life’ Indicator

Certain periods in life may be very intensive for individuals in terms of paid and unpaid work, as shown in Fig. 1. For young parents, unpaid work in the form of childcare may overlap with building up the career and/or studying, setting up their own apartment, taking up additional paid work to earn extra money that young families need, etc. We want to shed some light on this period of the ‘rush hour of life’ and investigate what other activities are sacrificed during this period. Providing more paid and unpaid work leaves us with less leisure and/or time for personal care. In order to quantify the RHOL, we follow the approach as commonly applied in economic theory and assume that 8 h out of the 24 h a day is spent on sleeping.^6^ The remaining 16 h per day can be devoted to ‘work time’ (encompassing paid work, unpaid work and education) and ‘free time’ which can be used for leisure activities or personal care activities including sleep above the 8 h. To identify the ‘rush hour of life’ indicator (or in short *RHOL indicator*) across gender and age groups, we calculate the percentage share of work time in total available time. The lowest possible value of RHOL indicator is 0%, implying that a person did not report any productive work at all. For example, a person could go hiking for a whole day on a weekend. On the other hand, the theoretical maximum of this indicator is 150%. This would happen in the extreme case when a person reported productive work for all 24 h per day, without even reporting any sleep, eating, etc. There are three such cases out of 50,825^7^, and in total there are 182 cases with RHOL indicators above 100. Thus, 99.7% of individuals have RHOL indicators between 0% and 100%. In our analysis, we are interested in the age groups at which the RHOL indicator exceeds 50%. A value of more than 50% means that the people’s average work time exceeds their free time, and we define those ages as the ‘rush hour of life’.

In Table 3, the RHOL is marked in italics and corresponds to the age groups with more than 480 min (8 h) of total work presented in Fig. 1. In Austria, this period lasts from age 20 to 54^8^ and there are no gender differences in the length or the intensity of rush hour, since during this prime age the total work load is more or less equally distributed among genders. For both genders, the rush hour of life is most intensive between ages 30 and 39. In Italy and Slovenia, the rush hour starts later, which is probably the effect of a delayed transition to adulthood, and there are clear gender differences in those countries. For women, the rush hour of life lasts longer than for men—about 10 years in Italy and about 5 years in Slovenia. Moreover, the rush hour of life is much more intensive for women than men. For men, the share of work time increases only to 54% in Italy and 57% in Slovenia, but for women it reaches 59% in Italy and even 63% in Slovenia. Thus, in Italy and Slovenia women sacrifice much more free time to provide all the paid and unpaid work presented earlier.

**Table 3:** Share of productive work time in total available time of 16 h per day

Age	Male			Female		
	Austria	Italy	Slovenia	Austria	Italy	Slovenia
10–14	36.4	35.3	31.2	36.6	36.9	34.1
15–19	44.7	36.1	40.4	45.1	40.0	42.1
20–24	*50.8*	38.7	40.9	*51.3*	43.7	45.3
25–29	*54.5*	46.2	*52.3*	*57.7*	*52.3*	*55.3*
30–34	*59.3*	*51.7*	*55.7*	*59.1*	*57.4*	*63.0*
35–39	*59.2*	*53.8*	*57.1*	*58.2*	*59.5*	*60.6*
40–44	*56.6*	*54.2*	*54.3*	*57.0*	*59.3*	*60.4*
45–49	*55.8*	*53.9*	*52.4*	*56.5*	*58.9*	*60.5*
50–54	*53.9*	*51.3*	49.0	*53.1*	*57.4*	*54.8*
55–59	46.3	44.4	43.2	44.7	*51.4*	47.3
60–64	30.3	34.2	35.8	37.0	46.8	47.9
65–69	30.0	27.9	35.3	38.4	43.8	44.5
70–74	28.9	25.7	29.2	36.9	40.9	39.6
75+	20.5	18.0	20.0	28.2	29.5	28.8

Source: Authors’ calculations on time use surveys in Austria (2008), Italy (2008) and Slovenia (2000)

In italics, we mark the ‘rush hour of life’—i.e. ages at which the share of work time is above 50%

Nevertheless, we should keep in mind that the RHOL values presented in this section are calculated as age- and sex-specific averages and thus provide us with a broad picture of gender differences with regard to free time within and between the three countries. In particular, different breadwinner arrangements among couples can impact on the amounts of free time available by gender. This is especially true for women, who traditionally bear the main responsibility for unpaid work activities. Population estimates for the three countries (Tables A4, A5 and A6 in Online Appendix) show the existence of relevant differences in the organisation of paid work among couples. Italy has the highest share of individuals in male-breadwinner arrangements across the three countries (23.8%) and the lowest share in dual-earner couples (24.1%). The opposite is true for Slovenia where individuals living in dual-earner couples make up the great majority of the total population (51.5%), whereas those in single-earner arrangements make up only 13% of the population. In Austria, 29.2% of the population lives in dual-earner couples, but the proportion of couples where he works full time and she works part time (18.4%), i.e. ‘modified-breadwinner couples’, exceeds that of male-breadwinner couples (14.3%). The share of population in modified-breadwinner couples is 9.5% in Italy, whereas it is extremely low in Slovenia (1.2%), so there working full time is the norm among those with a paid job. These compositional differences may significantly affect RHOL averages in the three countries. Results of the regression models presented in the next sections will help us to broaden our understanding of the RHOL by measuring its association with different breadwinner arrangements for men and women in the three countries.

## Linear Model for Analysing the RHOL

In order to quantify the relation between different breadwinner arrangements and the RHOL of men and women in the three countries, we use a linear regression model with interactive effects for gender. We limit our analysis to the 25–54 years age interval for which the RHOL indicator is above 50%. We distinguish the following types of couples’ breadwinner arrangements: male breadwinner, with man working full time and woman out of the labour force; dual earners, with both partners working full time; modified male-breadwinner model, with men working full time and women part time; and others, which include all remaining couples’ working arrangements plus individuals without a partner. Our typology of couples’ working arrangements (*COWAR*) does not include a separate category for female-breadwinner and modified female-breadwinner households. This choice was driven by the very small subsamples available in our data for such working arrangements. We also try to control for other characteristics that might affect time use. In the regression model, we therefore include the following explanatory variables for: age group (25–34, 35–44, 45–54); highest degree of education attained (high, medium, low); education of the partner (indicating whether the partner has higher education or not); presence of preschool children; presence of additional adults; marital status; and weekend days.

The dependent variable is the value of the RHOL indicator. We run the regression separately for the three countries. We implemented a standard linear model for Austria and Italy, assuming residual errors to be independent and normally distributed. To account for clustered data in the Slovenian sample, which includes two diaries for each respondent, we used a linear model accounting for correlated errors among observations made on the same subject.^9^ We are interested in the interaction of the presence of breadwinner arrangement variables with gender. For this reason, we allow all parameters to differ across genders by including an interaction term. Full results for the regression estimates of the RHOL indicator (together with the corresponding standard errors, sample size and relative composition of the population) are reported in Online Appendix (Tables A4, A5 and A6 for Austria, Italy and Slovenia, respectively).

We hypothesise breadwinner arrangements to be the main determinants of the RHOL for women but not of the one for men. For all three countries, we expect the values of the RHOL indicator to be higher for women in dual-earner couples and lower for those in male-breadwinner arrangements. We also focus on the presence of young children: we expect higher values of the RHOL for both mothers and fathers of preschool children. Nevertheless, we expect the effect to be greater for women than for men and, thus, the magnitude of gender differences in the RHOL to be higher.

## Determinants of the RHOL

As explained in the previous section, we limit our analysis to the age interval between 25 and 54 years for which the RHOL indicator is above 50% (Table 3). We conduct estimates separately for each country. Looking at the main effects of the *COWAR* (i.e. without interaction terms) for Austria in Table 4 shows that the RHOL indicator in male-breadwinner arrangements is statistically significantly lower than in the other arrangements. In particular, Austrians in male-breadwinner families face a RHOL that is 5.45 percentage points (p.p.) lower than in dual-earner arrangements, corresponding to approximately 52 min per day. Compared to couples where men work full time and women work part time, the RHOL is 4.26 p.p. (41 min per day) lower in male-breadwinner couples. The presence of preschool children is related to a 2.83 p.p. higher RHOL indicator. Among the set of control variables, education shows a statistically significant effect on the values of the indicator, with most highly educated Austrians facing a more intense RHOL. Looking at the crossed effect of gender with the *COWAR* typology reveals that different working arrangements are related to the RHOL of Austrian women but not to that of Austrian men. Austrian women in male-breadwinner arrangements are those reporting the least time stress: the value of their RHOL is reduced by 9.16 p.p. (almost 90 min per day) when compared to women in dual-earner couples and by 6.15 p.p. when compared to couples where he works full time and she works part time. Having preschool children is related to a higher RHOL for both genders: mothers and fathers report 2.94 p.p. and 2.73 p.p. higher RHOLs, respectively, compared to non-parents. Weekends are relevant in decreasing the RHOL for both genders, but the reduction is greater for men compared to women (32.61 p.p. and 24.47 p.p.). The interaction between education and gender shows a positive relation between education level and the RHOL.

**Table 4:** Linear model for Austria: simple and interactive effects by gender and covariate variables

	Austria—simple effects				Austria—gender interactive effects							
					Men				Women			
	Effect	Standard error	*t* value	Pr (> |t|)	Effect	Standard error	*t* value	Pr (> |t|)	Effect	Standard error	*t* value	Pr (> |t|)
Gender (ref.: men)												
Women	2.20	1.51	1.45	0.146								
Age group (ref.: 25–34)												
35–44	− 0.11	0.87	− 0.13	0.900	− 0.56	1.22	− 0.46	0.647	0.34	1.23	0.28	0.781
45–54	− 1.76	0.96	− 1.83	0.068	− 2.14	1.35	− 1.59	0.112	− 1.38	1.38	− 1.00	0.316
Education level (ref.: high)												
Medium	− 2.32	0.91	− 2.54	0.011	− 1.74	1.22	− 1.43	0.152	− 2.90	1.36	− 2.13	0.033
Low	− 6.67	1.51	− 4.43	< 0.001	− 6.19	2.28	− 2.72	0.007	− 7.15	1.97	− 3.63	< 0.001
Partner edu. (ref.: higher)												
Not/without a partner	− 1.18	0.51	− 0.94	0.345	− 0.37	2.01	− 0.19	0.852	− 1.99	1.49	− 1.33	0.183
COWAR (ref.: male breadwinner)												
Dual earner	5.45	1.14	4.76	< 0.001	1.73	1.65	1.05	0.294	9.16	1.58	5.78	< 0.001
Modified male breadwinner	4.26	1.21	3.52	< 0.001	2.36	1.73	1.36	0.173	6.15	1.69	3.64	< 0.001
Other	− 0.10	1.26	− 0.08	0.937	− 5.63	1.82	− 3.09	0.002	5.43	1.73	3.14	0.002
Married (ref.: not)												
Yes	− 0.19	0.90	− 0.22	0.830	− 0.53	1.29	− 0.41	0.683	0.14	1.24	0.11	0.909
Child 0–6 years (ref.: not)												
Yes	2.83	0.97	2.91	0.004	2.73	1.40	1.95	0.051	2.94	1.35	2.18	0.029
Additional adult (ref.: not)												
Yes	− 0.79	0.91	− 0.87	0.386	− 1.29	1.28	− 1.00	0.316	− 0.30	1.30	− 0.23	0.819
Weekend (ref.: not)												
Yes	− 28.54	0.75	− 38.23	< 0.001	− 32.61	1.06	− 30.75	< 0.001	− 24.47	1.05	− 23.28	< 0.001

Source	*df*	*F* Value	Pr > *F*
*Analysis of variance*			
Model	26	237.9	< 0.001
Error	4095		

Source: Authors’ calculations on Time Use Survey, Austria (2008)

A look at the main effects of the model for Italy (Table 5) highlights that couples’ working arrangements are significantly related to the levels of the RHOL indicator, with individuals in male-breadwinner arrangements reporting 6.05 p.p. and 4.15 p.p. lower values compared to those in dual- and modified-breadwinner couples. Preschool children confirm to be positively associated with the RHOL of couples, the values of the indicator being 5.41 p.p. higher compared to couples where small children are absent. Contrary to expectations, the interactive effects of the *COWAR* variable with gender show that breadwinner arrangements are associated with the rush hour indicator for men as well: the value of the RHOL for Italian men in male-breadwinner couples is 1.80 p.p. lower than in dual-earner ones, suggesting that men contribute more to domestic activities when women have a full-time job. A similar relation is found for women, although the magnitude of the effect is greater: Italian women face a 10.31 p.p. lower RHOL (that is a decrease of more than 1.5 h per day) when only the man works compared with being in a couple where both partners work full time. Lower values of the RHOL (6.73 p.p.) are also found for women in modified male-breadwinner arrangements. Having children is related to higher values of the RHOL for both genders although, once again, more so for women (6.65 p.p.) than for men (4.17 p.p.). Results for the control variables display a reverse relation of the rush hours to marital status by gender: being married is related to a lower value of the RHOL for men and a higher value for women. The presence of additional adults in the household lowers the working time of both genders, suggesting the relevance of intergenerational co-residence in Italy. More highly educated Italian women, contrary to Austrian ones, face a less intense RHOL. This result suggests that in Austria highly educated women are more likely to combine paid and unpaid work and therefore to experience a time squeeze, whereas in Italy highly educated women with good jobs and earnings may paradoxically work fewer hours in total, due to considerable reductions in domestic work that can be outsourced to men and the market. The rush hours are significantly reduced during weekends for both genders with men and women facing about 30.63 p.p. and 19.49 p.p. less RHOL per day (which corresponds to a reduction of approximately 5 and 3 h, respectively). It is worth noting that a number of socio-demographic characteristics are associated with the RHOL indicator in Italy. This is especially true for women for whom all the explicative variables introduced in our model reported to be statistically significant in determining its levels, indicating that there are numerous different factors affecting female time allocation to work and non-work activities in this Mediterranean country.

**Table 5:** Linear model for Italy—simple and interactive effects by gender and explicative variables

	Simple effects				Gender interactive effects							
					Men				Women			
	Effect	Standard error	*t* value	Pr (> |t|)	Effect	Standard error	*t* value	Pr (> |t|)	Effect	Standard error	*t* value	Pr (> |t|)
Gender (ref.: men)												
Women	5.04	0.66	7.63	< 0.001								
Age group (ref.: 25–34)												
35–44	1.58	0.38	4.10	< 0.001	0.11	0.55	0.19	0.846	3.05	0.53	5.70	< 0.001
45–54	2.57	0.43	5.96	< 0.001	0.76	0.62	1.23	0.220	4.39	0.61	7.26	< 0.001
Education level (ref.: high)												
Medium	0.99	0.45	2.19	0.028	− 0.82	0.66	− 1.24	0.214	2.79	0.61	4.59	< 0.001
Low	1.26	0.48	2.64	0.008	− 0.82	0.69	− 1.19	0.234	3.33	0.66	5.08	< 0.001
Partner edu. (ref.: higher)												
Not/without a partner	1.18	0.52	2.29	0.022	0.23	0.69	0.32	0.745	2.14	0.76	2.80	0.005
COWAR (ref.: male breadwinner)												
Dual earner	6.05	0.44	13.68	< 0.001	1.80	0.64	2.82	0.005	10.31	0.61	16.81	< 0.001
Modified male breadwinner	4.15	0.57	7.25	< 0.001	1.56	0.82	1.90	0.057	6.73	0.79	8.47	< 0.001
Other/without a partner	− 2.84	0.53	− 5.38	< 0.001	− 8.79	0.79	− 11.17	< 0.001	3.12	0.70	4.44	< 0.001
Married (ref.: not)												
Yes	0.66	0.48	1.39	0.165	− 1.93	0.71	− 2.70	0.007	3.26	0.64	5.10	< 0.001
Child 0–6 years (ref.: not)												
Yes	5.41	0.40	13.44	< 0.001	4.17	0.58	7.20	< 0.001	6.65	0.56	11.90	< 0.001
Additional adult (ref.: not)												
Yes	− 3.06	0.34	− 8.87	< 0.001	− 4.02	0.49	− 8.16	< 0.001	− 2.10	0.48	− 4.34	< 0.001
Weekend (ref.: not)												
Yes	− 25.06	0.33	− 76.66	< 0.001	− 30.63	0.46	− 66.11	< 0.001	− 19.49	0.46	− 42.24	< 0.001

Source	*df*	*F* value	Pr > *F*
*Analysis of variance*			
Model	26	1199	< 0.001
Error	16,807		

Source: Authors’ calculations on Time Use Survey, Italy (2008)

Finally, looking at the main effects of the model for Slovenia (Table 6), different breadwinner typologies show a significant relation to the levels of the RHOL indicator only when individuals in male-breadwinner couples are compared to those in dual-earner ones (with the former facing a RHOL that is 4.18 p.p. or about 40 min lower). The presence of young children is related to a 4.40 p.p. higher RHOL. Similar to Austria, the interactive effects of the COWAR variable with gender reveal that the levels of free time available for Slovenian men are not significantly affected by breadwinner arrangements. On the other hand, being in a couple where only the man works in the market is associated with lower values of the RHOL indicator for Slovenian women. The magnitude of the reduction is similar when comparing women in male-breadwinner with those in dual-earner and modified male-breadwinner couples (8.82 p.p. and 9.02 p.p. less, respectively), although statistical significance is lower for the latter case due to the small prevalence of this working arrangement in Slovenia. Having preschool children is related to a significantly higher RHOL of both men and women. However, surprisingly, the effect of children is greater for fathers than for mothers. This is probably due to the fact that Slovenian men engage more in domestic work (and especially in care activities) only when they are fathers of young children. Thus, the additive effect of having children on their levels of unpaid work is higher than for women who are traditionally committed to domestic activities. As for the remaining control variables, it is worth noting that the relation between marital status and the RHOL for men and women is similar to the one found for Italy. Van der Lippe and colleagues (2010) suggest that while single persons will assume all domestic responsibilities for themselves alone, being married implies the formation of a two-person household and thus more time needed for housework (even though the effect is attenuated by the presence of economies of scale). Due to gender specialisation, the female partner is likely to take the responsibility for most of the housework. Therefore, large amounts of unpaid work are shifted from men to women when they are married. This shift is likely to combine with the already high levels of female unpaid work in Italy and of female paid work in Slovenia, generating a more intense RHOL for married women in these two countries compared with their single counterparts. On the other hand, being married shows no relation with the RHOL in Austria which may be explained by diffusion of part-time work among Austrian women ensuring relatively lower levels of total work, compared to male-breadwinner and dual-earner arrangements. Similar to the other two countries, men report a greater reduction in the RHOL during weekends than women.

**Table 6:** Linear model for Slovenia—simple and interactive effects by gender and covariate variables

	Simple effects				Gender interactive effects							
					Men				Women			
	Effect	Standard error	*t* value	Pr (> |t|)	Effect	Standard error	*t* value	Pr (> |t|)	Effect	Standard error	*t* value	Pr (> |t|)
Gender (ref.: men)												
Women	3.53	1.67	2.11	0.035								
Age group (ref.: 25–34)												
35–44	0.35	0.80	0.43	0.666	0.04	1.16	0.04	0.971	0.65	1.11	0.59	0.557
45–54	− 1.79	0.88	− 2.03	0.042	− 3.23	1.29	− 2.52	0.012	− 0.36	1.21	− 0.29	0.770
Education level (ref.: high)												
Medium	1.49	0.86	1.73	0.083	1.83	1.31	1.39	0.164	1.16	1.12	1.04	0.300
Low	2.10	0.87	2.41	0.016	2.49	1.31	1.91	0.056	1.71	1.16	1.47	0.141
Partner edu. (ref.: higher)												
Not/without a partner	0.96	0.79	1.21	0.225	0.90	1.11	0.81	0.417	1.01	1.12	0.90	0.366
COWAR (ref.: male breadwinner)												
Dual earner	4.18	0.92	4.55	< 0.001	− 0.45	1.30	− 0.35	0.727	8.82	1.31	6.75	< 0.001
Modified male breadwinner	4.20	2.73	1.54	0.124	− 0.62	3.86	− 0.16	0.873	9.02	3.86	2.34	0.020
Other/without a partner	− 5.08	1.10	− 4.60	< 0.001	− 12.76	1.65	− 7.73	< 0.001	2.60	1.47	1.77	0.077
Married (ref.: not)												
Yes	− 1.33	0.94	− 1.43	0.154	− 5.21	1.41	− 3.70	< 0.001	2.54	1.23	2.06	0.040
Child 0–6 years (ref.: not)												
Yes	4.40	0.87	5.04	< 0.001	5.26	1.24	4.24	< 0.001	3.54	1.23	2.88	0.004
Additional adult (ref.: not)												
Yes	1.49	0.67	2.23	0.026	1.77	97	1.83	0.068	1.20	0.91	1.32	0.188
Weekend (ref.: not)												
Yes	− 23.13	0.53	− 43.69	< 0.001	− 25.65	0.76	− 33.87	< 0.001	− 20.60	0.74	− 27.85	< 0.001

Source	*df*	*F* value	Pr > *F*
*Analysis of variance*			
Model	26	249.2	< 0.001
Error	3.127		

Source: Authors’ calculations on Time Use Survey, Slovenia (2000)

Summarising, couples’ breadwinner arrangements are significantly related to their RHOL values. Women in dual-earner couples work longer hours in total and have less free time available. Those in male-breadwinner arrangements report the lowest RHOL values. As expected, couples’ working arrangements were not statistically significant in explaining the RHOL of men in Austria and Slovenia. However, Italian men in dual-earner and modified male-breadwinner couples did report higher levels of time devoted to work.

In all three countries, dual-earner arrangements significantly affect leisure inequality, with women in full-time employed couples working longer hours and thus reporting a significantly higher RHOL, compared to men in a similar situation. Having a preschool child is associated with more intense rush hours for both genders. Unexpectedly, the magnitude of the effect was greater for mothers than fathers only in Italy, whereas gender distribution was equal in Austria. Slovenia reported evidence of an opposite situation with fathers of young children increasing their overall working time more than mothers. Thus, Italy was the only country where the net effect of having young children was to exacerbate gender inequalities in terms of free time available for women. These results seem to be in line with Gauthier et al. (2004) who in a study on parental time over 16 industrialised countries showed that, despite the existence of a general trend towards increasing fathers’ time with children, there are still considerable cross-nation variations regarding the gender gap in time for childcare.

The male and female values of the RHOL are fairly homogeneous in Austria and Slovenia after controlling for the different breadwinner arrangements and weekend days, whereas a greater number of factors are associated with time allocation to work and leisure in Italy, especially for women.

## Conclusions

In this paper, we estimate the age-specific involvement in paid work and different forms of unpaid work for both sexes. Age- and gender-specific work activities are very different across countries. In Austria, the load of paid and unpaid work together is similar for both genders, whereas in Slovenia and Italy it is much higher for women than for men. However, the composition of women’s workload in Italy and Slovenia is inherently different: Slovenian women are strongly involved in paid work, which is a characteristic of ex-socialistic countries. After they finish with the work in the formal sector, they work another ‘shift’ at home, which is in line with the ‘double shift’ hypothesis known from the literature. The consequence is a high workload of above 550 min per day during the entire working period. Italian women use little time for paid work, but they spend much more time on unpaid work than women in Slovenia or Austria. The average time devoted to total work by Italian women in working age is about 550 min at a similar level as for women in Austria and much higher than the time Italian men devote to work. Austria shows a more traditional pattern regarding gender work division—men are more involved in paid work and women more in unpaid work.

A novelty in this paper is the development of the indicator of the rush hour of life (RHOL), which allows for measures of the time squeeze by gender over the life course. A limit of this approach can be represented by the objective nature of the indicator that allows no evaluation of individual feelings and well-being, i.e. some people may feel happy being overworked, whereas others may feel very stressed. Similarly, the border between work and leisure may be somewhat blurred. One future direction of this research is to combine objective measures of time scarcity and the use of time with information on individual subjective feelings and perceptions.

Nevertheless, this study contributes to shedding light on periods of time pressure during life and to gaining important insights into the division of market and non-market work across genders and different institutional contexts. Our results reveal the existence of relevant differences among men and women in the three countries. In Austria, there are no gender differences in the length or the intensity of the rush hour since during prime age the total work load is about equally distributed among genders, although men are more specialised in paid work and women in unpaid work. On the other hand, in Italy and Slovenia the RHOL is more intense and lasts longer for women than for men. Nevertheless, breadwinner arrangements considerably differ among Austria, Italy and Slovenia and this can considerably affect the amount of total work and levels of free time available for men and women in these three countries. Thus, we move a step forward by attempting to quantitatively assess the association between different breadwinner arrangements and the RHOL indicator for men and women. To do so, we run a linear regression model for each country with interactive effects for gender. In addition to a typology of couples’ working arrangements, we include a number of explanatory variables that may affect the RHOL indicator. Of particular interest for us is the presence of preschool children. Results confirmed our hypothesis that couples’ work arrangements are relevant for understanding differences in the RHOL of women but not that of men. Nevertheless, an exception to this was found for Italian men in dual-earner couples who reported higher RHOL values compared to those in households where women did not have a paid job. Results for all countries yielded evidence for a dual burden of women in full-time working couples, who reported the highest RHOL values. By contrast, women in male-breadwinner arrangements are those facing the lowest level of time squeeze.

The emergence of two-income families and the strategies adopted by couples for coping with family and job responsibilities have received considerable attention from scholars in recent decades. Work–family reconciliation has firmly entered the agenda of contemporary welfare states. Role strain and work overload have been identified as major problems for dual-earner families and particularly for women who continue to be mainly responsible for household and family care even when they have a paid job (Anderson and Leslie 1991). In a similar panorama, single-earner families may represent a strategy to cope with the family–work conflict, especially in countries with lacking or inadequate reconciliation policies. Also other factors, in addition to policies and services available for families, may influence a couple’s decision towards a single-income arrangement. Several studies have provided evidence for a ‘motherhood penalty’ in employment: mothers are disadvantaged in finding a job and being perceived as competent workers, and often earn less than childless colleagues with similar qualifications (Budig and England 2001). Families’ working arrangements also depend on structural constraints of the labour market (such as availability of jobs) and result from cultural norms and limited human capital (Moen and Yu 2000). Couples’ choices in this regard can be taken less or more voluntarily. However, understating the extent to which such choices are explicit or not is not the purpose of this paper. Our purpose is to measure and analyse total work time and the related scarcity of free time over the life course across genders in different institutional and family contexts.

Broadening the understanding of the RHOL is fundamental for the development of effective work–family reconciliation policies which are, in turn, inherently relevant for contemporary welfare states. Abstaining from having children or from having a career is potential strategies to deal with the family–work conflict, both of which can result in high costs for individuals and society as whole. Reforms of the welfare system should take into account their effects on the time shortage of the population in these age groups which play such an essential role in forming the human capital of the society and in providing the funding for the social systems. With the emergence of ageing societies, increased female participation in the labour market and human capital is key to ensuring the future sustainability of welfare states.

## Electronic supplementary material

Below is the link to the electronic supplementary material.

Supplementary material 1 (PDF 364 kb)

## Footnotes

1 Diaries for children in preschool ages were filled in by their parents.

2 In both countries, time use information was collected during a single day using one 24-h time diary.

3 The sample size of the population aged 10 + for the three countries is reported in Table A1 in Online Appendix.

4 ‘Voluntary work represents unpaid activities such as work for and through charity and other non-profit organisations as well as informal help provided to other households—for example food management, household upkeep, gardening, shopping.

5 Breaks usually refer to time for lunch and coffee during work. Doubts can arise whether this time should be considered as paid work or personal care/leisure. However, issues in the reporting and codification of time for breaks in our data do not allow to subtract lunch breaks from paid work or to define a threshold for lunch breaks. Nevertheless, given the relatively low amounts of time reported as spent on breaks, the inclusion of this activity in the category of paid work does not significantly affect our results. This view seems to be confirmed by the fact that our classification of lunch breaks is a common approach in time use studies (e.g. Apps and Rees 2005).

6 For example, Ehrenberg and Smith (2016) present decisions of an individual about allocating 16 h per day between hours of work and hours of leisure, whereas the remaining 8 h per day is assumed to be used for sleeping and other ‘maintenance activities’ and is therefore excluded from the analysis.

7 This number is the overall sample size of individuals aged 10 + years in the three countries.

8 We list the lower boundary of the first and the upper boundary of the last 5-year age group at which the ratio of free time to work is below 1.

9 We used the SAS GLM procedure to fit the regression model for Austria and Italy, whereas, for Slovenia, we used SAS PROC MIXED which provides a variety of covariance structures and fits the selected structure by using the method of restricted maximum likelihood (Vonesh 2012). Both procedures allow to compute least square mean (LS-means) estimates of the RHOL together with their standard errors for each effect listed in the model.
