# Supplementary material for: A Quantitative Assessment of the Rush Hour of Life in Austria, Italy and Slovenia
Source: Eur J Popul. 2018 Nov 21;35(4):751–76. doi: 10.1007/s10680-018-9502-4 (PMC6797675; doi:10.1007/s10680-018-9502-4)
Supplement: Supplementary file 1 — Supplementary material 1 (PDF 364 kb) [file 10680_2018_9502_MOESM1_ESM.pdf]

On-line Appendix

Table A1 Sample Size by Country, Gender and Age Group (individuals aged 10+)

Age Group	Austria			Italy			Slovenia		
	Men	Women	**Total**	Men	Women	**Total**	Men	Women	**Total**
10-14	219	194	**413**	996	899	**1 ,895**	170	149	**319**
15-19	207	221	**428**	1,017	988	**2 ,005**	254	260	**514**
20-24	211	227	**438**	999	937	**1 ,936**	305	364	**669**
25-29	229	305	**534**	998	1 ,033	**2 ,031**	257	281	**538**
30-34	269	343	**612**	1,244	1 ,382	**2 ,626**	231	226	**457**
35-39	350	426	**776**	1,503	1 ,539	**3 ,042**	209	253	**462**
40-44	358	427	**785**	1,678	1 ,736	**3 ,414**	231	295	**526**
45-49	345	429	**774**	1,493	1 ,533	**3 ,026**	293	342	**635**
50-54	291	349	**640**	1,296	1 ,398	**2 ,694**	254	279	**533**
55-59	292	347	**639**	1,284	1 ,338	**2 ,622**	199	184	**383**
60-64	272	317	**589**	1,149	1 ,249	**2 ,398**	169	202	**371**
65-69	258	327	**585**	1,098	1 ,289	**2 ,387**	142	151	**293**
70-74	181	221	**402**	991	1 ,167	**2 ,158**	99	127	**226**
75+	237	380	**617**	1 ,618	2 ,551	**4 ,169**	99	165	**264**
**Total**	**3 ,719**	**4 ,513**	**8 ,232**	**17 ,364**	**19 ,039**	**36 ,403**	**2 ,912**	**3 ,278**	**6 ,190**

Source: Authors' calculations on Time Use Surveys Austria (2008), Italy (2008), Slovenia (2000)

Table A2 Standard Error of Mean by activities and age groups (minutes per day)

	Austria - Men					Austria - Women				
	**10-24**	**25-39**	**40-59**	**60+**	**Total**	**10-24**	**25-39**	**40-59**	**60+**	**Total**
Housework	*4*	*4*	*4*	*5*	**3**	*4*	*4*	*4*	*4*	***2***
Childcare	*1*	*2*	*1*	*1*	**2**	*2*	*4*	*1*	*1*	***2***
Adultcare	*0*	*0*	*0*	*1*	***1***	*0*	*0*	*1*	*1*	***1***
Voluntary	*1*	*1*	*1*	*2*	**3**	*1*	*1*	*1*	*1*	***2***
Paid Work	11	11	9	4	**7**	10	9	7	2	**6**
Education	10	2	1	1	**8**	10	2	1	0	**7**
Personal Care	7	5	4	4	**3**	6	4	3	4	**2**
Leisure	8	7	5	6	**3**	7	5	4	4	**3**
Other/Unknown	1	1	1	1	**1**	0	0	1	0	**1**

	Italy - Men					Italy - Women				
	**10-24**	**25-39**	**40-59**	**60+**	**Total**	**10-24**	**25-39**	**40-59**	**60+**	**Total**
Housework	*1*	*2*	*2*	*2*	***1***	*2*	*3*	*2*	*2*	***1***
Childcare	*0*	*1*	*1*	*0*	***0***	*1*	*2*	*1*	*0*	***1***
Adultcare	*0*	*0*	*0*	*0*	***0***	*0*	*0*	*0*	*0*	***0***
Voluntary	*0*	*1*	*1*	*1*	***0***	*1*	*1*	*0*	*1*	***0***
Paid Work	3	5	4	2	**2**	3	4	3	1	**1**
Education	4	1	0	0	**1**	4	1	0	0	**1**
Personal Care	3	2	2	2	**1**	3	2	2	2	**1**
Leisure	3	3	3	3	**2**	3	3	2	2	**1**
Other/Unknown	0	0	0	0	**0**	0	0	0	0	**0**

	Slovenia - Men					Slovenia - Women				
	**10-24**	**25-39**	**40-59**	**60+**	**Total**	**10-24**	**25-39**	**40-59**	**60+**	**Total**
Housework	*3*	*4*	*4*	*5*	***2***	*3*	*4*	*4*	*4*	***2***
Childcare	*0*	*2*	*1*	*1*	***1***	*1*	*3*	*1*	*1*	***1***
Adultcare	*0*	*1*	*0*	*1*	***0***	*0*	*0*	*0*	*1*	***0***
Voluntary	*1*	*1*	*1*	*2*	***1***	*1*	*1*	*1*	*1*	***0***
Paid Work	5	8	6	4	**4**	5	6	6	2	**3**
Education	6	2	1	0	**2**	6	2	1	0	**2**
Personal Care	4	3	3	3	**2**	4	3	3	3	**2**
Leisure	6	5	4	5	**3**	5	4	4	4	**2**
Other/Unknown	1	1	1	1	**0**	1	1	1	1	**0**

Source: Authors' calculations on Time Use Surveys Austria (2008), Italy (2008), Slovenia (2000)

Table A3 Share of providers and minutes per provider by sub-groups of unpaid work

	Austria - Men					Austria - Women				
	**10-24**	**25-39**	**40-59**	**60+**	**Total**	**10-24**	**25-39**	**40-59**	**60+**	**Total**
	Share of providers					Share of providers				
Housework	56	74	76	89	**74**	71	95	97	97	**92**
Childcare	3	31	17	6	**15**	9	51	20	9	**22**
Adultcare	1	2	4	3	**3**	2	4	8	5	**5**
Voluntary	3	4	6	9	**6**	4	3	5	7	**5**
	Minutes per provider					Minutes per provider				
Housework	92	126	166	230	**162**	122	206	261	305	**240**
Childcare	88	106	78	81	**93**	147	182	107	101	**149**
Adultcare	49	64	57	74	**62**	58	48	71	74	**67**
Voluntary	125	149	127	117	**127**	101	135	99	85	**100**

	Italy - Men					Italy - Women				
	**10-24**	**25-39**	**40-59**	**60+**	**Total**	**10-24**	**25-39**	**40-59**	**60+**	**Total**
	Share of providers					Share of providers				
Housework	39	64	72	83	**67**	68	93	98	94	**91**
Childcare	1	24	21	7	**15**	5	48	27	8	**23**
Adultcare	2	3	6	8	**5**	3	7	13	7	**8**
Voluntary	2	3	4	9	**5**	4	6	9	10	**8**
	Minutes per provider					Minutes per provider				
Housework	73	123	157	207	**157**	124	327	351	349	**319**
Childcare	53	93	81	94	**87**	164	167	98	109	**134**
Adultcare	110	65	66	86	**76**	59	34	56	83	**59**
Voluntary	137	123	109	125	**121**	105	86	96	103	**98**

	Slovenia - Men					Slovenia - Women				
	**10-24**	**25-39**	**40-59**	**60+**	**Total**	**10-24**	**25-39**	**40-59**	**60+**	**Total**
	Share of providers					Share of providers				
Housework	66	77	83	88	**78**	80	96	98	97	**94**
Childcare	5	31	12	13	**15**	9	54	19	15	**24**
Adultcare	3	5	7	4	**5**	5	9	8	5	**7**
Voluntary	6	6	8	10	**7**	4	5	7	5	**5**
	Minutes per provider					Minutes per provider				
Housework	105	157	204	246	**182**	125	234	312	346	**267**
Childcare	48	91	60	78	**78**	138	135	69	84	**113**
Adultcare	72	58	52	71	**60**	52	38	54	87	**55**
Voluntary	160	156	165	127	**152**	89	107	84	99	**94**

Source: Authors' calculations on Time Use Surveys Austria (2008), Italy (2008), Slovenia (2000)

Table A4 Austria: Sample size, relative composition of the population, RHOL Estimate and Standard Errors by Gender and Covariate Variables

	All				Men				Women			
	Sample Size	Population %	RHOL Estimate	Standard Error	Sample Size	Population %	RHOL Estimate	Standard Error	Sample Size	Population %	RHOL Estimate	Standard Error
**All**	**4,121**	**100.0**	**-**	**-**	**1,842**	**100.0**	**50.14**	**1.16**	**2,279**	**100.0**	**52.34**	**0.97**
**Age group**												
25-34	1,146	30.2	51.86	0.90	498	29.7	51.04	1.35	648	30.8	52.69	1.19
35-44	1,561	37.4	51.76	0.89	708	37.7	50.48	1.33	853	37.0	53.03	1.18
45-54	1,414	32.4	50.10	0.96	636	32.6	48.90	1.43	778	32.2	51.31	1.27
**Education level**												
high	737	17.7	54.24	1.11	378	20.1	52.79	1.60	359	15.4	55.69	1.56
medium	2,938	71.3	51.92	0.79	1,331	72.8	51.04	1.22	1,607	69.7	52.79	1.01
low	446	11.0	47.57	1.19	133	7.1	46.59	1.90	313	14.8	48.54	1.44
**Education partner**												
higher	529	12.4	51.83	1.19	137	7.4	50.33	1.92	392	17.5	53.34	1.41
not/without a partner	3,592	87.6	50.65	0.71	1,705	92.6	49.95	1.02	1,887	82.5	51.35	0.99
**Cowar**												
dual earner	1,233	29.2	54.29	0.94	569	29.9	52.26	1.39	664	28.5	56.32	1.27
modified male breadwinner	823	18.4	53.10	1.07	377	18.9	52.89	1.57	446	17.9	53.30	1.47
male breadwinner	644	14.3	48.84	1.13	304	13.4	50.52	1.72	340	15.3	47.16	1.46
other/without a partner	1,421	38.0	48.74	0.95	592	37.8	44.89	1.43	829	38.3	52.59	1.27
**Married**												
yes	2,384	55.1	51.14	0.78	1,053	53.1	49.88	1.20	1,331	57.0	52.41	0.99
not	1,737	44.9	51.34	0.97	789	46.9	50.41	1.45	948	43.0	52.27	1.29
**Child 0-6 years**												
yes	1,019	21.3	52.66	1.01	461	20.9	51.50	1.50	558	21.7	53.81	1.36
not	3,102	78.7	49.83	0.77	1,381	79.1	48.78	1.20	1,721	78.3	50.87	0.96
**Additional adult**												
yes	699	17.2	50.84	1.04	321	17.8	49.50	1.53	378	16.7	52.19	1.40
not	3,422	82.8	51.64	0.70	1,521	82.2	50.78	1.09	1,901	83.3	52.49	0.87
**Weekend**												
yes	1,001	29.0	36.97	0.90	470	28.9	33.84	1.35	531	29.2	40.11	1.19
not	3,120	71.0	65.51	0.78	1,372	71.1	66.44	1.20	1,748	70.8	64.58	1.00

Source: Authors' calculations on Time Use Survey, Austria (2008)

Table A5 Italy: Sample size, relative composition of the population, RHOL Estimate and Standard Errors by Gender and Covariate Variables

	All				Men				Women			
	Sample Size	Population %	RHOL Estimate	Standard Error	Sample Size	Population %	RHOL Estimate	Standard Error	Sample Size	Population %	RHOL Estimate	Standard Error
**All**	**16,833**	**100.0**	**-**	**-**	**8,212**	**100.0**	**48.65**	**0.45**	**8,621**	**100.0**	**53.69**	**0.48**
**Age group**												
25-34	4,657	29.3	49.79	0.40	2,242	29.2	48.36	0.56	2,415	29.5	51.21	0.56
35-44	6,456	39.0	51.36	0.38	3,181	39.8	48.47	0.52	3,275	38.2	54.26	0.54
45-54	5,720	31.7	52.36	0.44	2,789	31.1	49.12	0.60	2,931	32.3	55.61	0.63
**Education level**												
high	2,479	14.7	50.42	0.50	1,057	13.1	49.20	0.73	1,422	16.2	51.65	0.70
medium	7,429	44.3	51.41	0.36	3,559	43.7	48.38	0.50	3,870	44.9	54.44	0.53
low	6,925	41.0	51.68	0.35	3,596	43.2	48.38	0.46	3,329	38.8	54.98	0.52
**Education partner**												
higher	1,889	10.8	50.58	0.53	1,107	12.8	48.54	0.71	782	8.9	52.62	0.79
not/without a partner	14,944	89.2	51.76	0.26	7,105	87.2	48.76	0.38	7,839	91.1	54.76	0.36
**Cowar**												
dual earner	3,946	24.1	55.39	0.43	1,891	23.3	51.81	0.59	2,055	24.8	58.97	0.62
modified male breadwinner	1,760	9.5	53.48	0.58	852	9.2	51.57	0.82	908	9.8	55.38	0.81
male breadwinner	3,884	23.8	49.33	0.46	1,853	23.0	50.01	0.66	2,031	24.6	48.65	0.63
other/without a partner	7,243	42.6	46.49	0.40	3,616	44.5	41.22	0.58	3,627	40.7	51.77	0.57
**Married**												
yes	10,182	60.5	51.50	0.32	4,721	56.7	47.69	0.45	5,461	64.3	55.32	0.47
not	6,651	39.5	50.84	0.48	3,491	43.3	49.62	0.68	3,160	35.7	52.06	0.67
**Child 0-6 years**	0											
yes	3,928	24.0	53.88	0.44	1,898	23.5	50.74	0.61	2,030	24.4	57.02	0.63
not	12,905	76.0	48.47	0.33	6,314	76.5	46.57	0.45	6,591	75.6	50.37	0.47
**Additional adult**												
yes	7,196	42.6	49.64	0.41	3,477	42.3	46.64	0.57	3,719	42.8	52.64	0.58
not	9,637	57.4	52.70	0.34	4,735	57.7	50.66	0.46	4,902	57.2	54.74	0.49
**Weekend**												
yes	10,646	28.6	38.64	0.40	5,163	28.6	33.33	0.55	5,483	28.6	43.95	0.57
not	6,187	71.4	63.70	0.34	3,049	71.4	63.97	0.46	3,138	71.4	63.43	0.49

Source: Authors' calculations on Time Use Survey, Italy (2008)

Table A6 Slovenia: Sample size, relative composition of the population, RHOL Estimate and Standard Errors by Gender and Covariate Variables

	All				Men				Women			
	Sample Size	Population	RHOL Estimate	Standard Error	Sample Size	Population	RHOL Estimate	Standard Error	Sample Size	Population	RHOL Estimate	Standard Error
**All**	**3 ,151**	**100.0**	-	-	**1,475**	**100.0**	50.14	1.16	**1 ,676**	**100**	52.34	0.97
**Age group**												
25-34	995	31.7	52.96	0.89	488	31.4	51.78	1.29	507	31.9	54.15	1.24
35-44	988	33.9	53.31	0.95	440	33.4	51.82	1.34	548	34.4	54.79	1.34
45-54	1 ,168	34.4	51.17	1.03	547	35.1	48.54	1.48	621	33.7	53.79	1.44
**Education level**												
high	504	17.6	51.28	1.07	189	14.2	49.27	1.58	315	20.8	53.29	1.44
medium	1 ,118	36.9	52.77	0.91	498	35.6	51.10	1.30	620	38.1	54.44	1.28
low	1 ,529	45.5	53.38	0.88	788	50.1	51.77	1.21	741	41.1	55.00	1.27
**Education partner**												
higher	551	18.2	52.00	1.03	281	20.3	50.26	1.45	270	16.2	53.74	1.46
not/without a partner	2 ,600	81.8	52.96	0.81	1 ,194	79.7	51.16	1.16	1 ,406	83.8	54.75	1.13
**Cowar**												
dual earner	1 ,513	51.5	55.84	0.67	717	52.9	53.72	0.92	796	50.1	57.95	0.96
modified male breadwinner	42	1.2	55.85	2.64	20	1.2	53.55	3.75	22	1.2	58.16	3.73
male breadwinner	446	13.0	51.65	0.98	207	13.2	54.17	1.40	239	12.8	49.13	1.38
other/without a partner	1 ,150	34.3	46.57	0.74	531	32.7	41.41	1.13	619	35.8	51.73	0.96
**Married**												
yes	2 ,276	72.9	51.81	0.82	1 ,015	71.0	48.11	1.17	1 ,261	74.8	55.51	1.14
not	875	27.1	53.15	1.08	460	29.0	53.32	1.56	415	25.2	52.97	1.49
**Child 0-6 years**												
yes	548	20.7	54.68	1.03	265	21.4	53.34	1.45	283	20.0	56.01	1.45
not	2 ,603	79.3	50.28	0.85	1 ,210	78.6	48.08	1.22	1 ,393	80.0	52.48	1.19
**Additional adult**												
yes	2 ,191	61.5	53.22	0.89	1 ,032	61.7	51.60	1.27	1 ,159	61.3	54.85	1.25
not	960	38.5	51.73	0.91	443	38.3	49.83	1.29	517	38.7	53.64	1.28
**Weekend**												
yes	1 ,543	28.6	40.91	0.92	722	28.6	37.89	1.30	821	28.6	43.94	1.29
not	1 ,609	71.4	64.04	0.83	754	71.4	63.54	1.19	855	71.4	64.54	1.18

Source: Authors' calculations on Time Use Survey, Slovenia (2000)
